# Inheritance of vernalization memory at *FLOWERING LOCUS C* during plant regeneration

**DOI:** 10.1093/jxb/erx154

**Published:** 2017-05-11

**Authors:** Miyuki Nakamura, Lars Hennig

**Affiliations:** Department of Plant Biology and Linnean Center for Plant Biology, Swedish University of Agricultural Sciences, Uppsala, Sweden

**Keywords:** *Arabidopsis thaliana*, chromatin, development, epigenetic inheritance, phase transition, shoot regeneration

## Abstract

Specific gene states can be transmitted to subsequent cell generations through mitosis involving particular chromatin (epigenetic) states. During reproduction of plants and animals, however, most epigenetic states are reset to allow development to start anew. Flowering is one of the critical developmental steps by which plants acquire their reproductive capacity. This phase transition is controlled by environmental signals and autonomous regulation. The *FLOWERING LOCUS C (FLC*) gene is a flowering repressor that is epigenetically silenced after long-term exposure to cold, ensuring flowering in the spring season. In *Arabidopsis thaliana,* epigenetically silenced *FLC* expression is reset during sexual reproduction. Plants have a remarkable potential to regenerate from somatic cells. However, little is known about whether the regeneration process is similar to sexual reproduction in terms of affecting chromatin states. Here, we tested whether *FLC* silencing is reset during *in vitro* regeneration. Transcriptional repression and high H3K27me3 at *FLC* were both stably transmitted, resulting in early flowering in regenerated shoots. Thus, the silenced epigenetic state of *FLC* is reset only during sexual reproduction and not during *in vitro* regeneration. In contrast, the active epigenetic state of *FLC* was only partially maintained through *in vitro* reproduction, suggesting that regeneration causes stochastic *FLC* silencing.

## Teaser

Histone H3K27me3-mediated silencing of the flowering repressor *FLOWERING LOCUS C* (*FLC*) was maintained throughout *in vitro* shoot regeneration. Furthermore, the regeneration process positively influenced the H3K27me3 mark on *FLC*.

## Introduction

Some gene activity states established during development are transmitted to subsequent cell generations through mitosis, often involving particular chromatin (epigenetic) states. Polycomb Group (PcG) proteins are a well-known group of chromatin modifiers that are involved in the maintenance of developmental gene repression in animals and plants, usually through trimethylation at lysine 27 of histone H3 (H3K27me3) at target genes (Geisler and Paro, 2015; Mozgova and Hennig, 2015).

The flowering repressor *FLOWERING LOCUS C* (*FLC*) in *Arabidopsis thaliana* is a well-studied plant PcG protein target (Bastow *et al.*, 2004; Sung and Amasino 2004). FLC delays flowering by repressing activators of flowering such as *FLOWERING TIME* (*FT*) (Helliwell *et al.*, 2006; Searle *et al.*, 2006; Geisler and Paro, 2015). The prolonged cold of winter leads to reduced *FLC* expression (Sheldon *et al.*, 2000; Mozgova and Hennig, 2015). Because *FLC* is kept inactive by PcG proteins even when temperatures rise again (Gendall *et al.*, 2001; Helliwell *et al.*, 2006), flowering can occur rapidly, allowing plants to use the favorable conditions of spring for reproduction. The increase in competence to flower brought about by prolonged exposure to cold is called vernalization (Chouard, 1960).

During reproduction, most epigenetic states on genes are reset to allow development to start anew (Paszkowski and Grossniklaus, 2011). *FLC* expression, for instance, is reset during reproduction and each generation has to be vernalized to repress *FLC* for accelerating flowering (Sheldon *et al.*, 2008; Yun *et al.*, 2011). In addition to sexual reproduction, plants have a remarkable potential to regenerate from somatic cells. This regeneration often involves a transient dedifferentiation into callus. Although *in vitro* plant regeneration has been studied for decades, little is known about whether epigenetic marks are transmitted through callus. For instance, it is not known whether epigenetic states are reset, as occurs during sexual reproduction, or whether they are transmitted to the *in vitro* progeny. Older observations in the biennial plant honesty (*Lunaria biennis*), the Arabidopsis late-flowering ecotype *Pitzal*, and the perennial weed chicory (*Cichorium intybus L.*) suggest that the vernalized state is maintained after *in vitro* culture (Wellensiek, 1961, 1962; Burn *et al.*, 1993; Demeulemeester and De Proft, 1999), but detailed molecular studies are not available. For instance, it has remained unknown whether *FLC* expression and H3K27me3 are affected by *in vitro* regeneration.

Here, we tested whether *FLC* silencing is inherited through *in vitro* regeneration. We found that regenerated shoots from vernalized plants flowered earlier than those from non-vernalized plants. Repression of *FLC* and high H3K27me3 at *FLC*, which were induced by vernalization, were stably inherited and not generally reset. Interestingly, some regenerated shoots from non-vernalized plants flowered earlier than the other plants in this group. Moreover, regeneration of shoots led to a moderate increase in H3K27me3 at *FLC* even without vernalization. Thus, callus induction can affect the abundance of H3K27me3 at *FLC*, and increased H3K27me3 at *FLC* may be sustained even in regenerated plants.

## Materials and methods

### Plant materials and growth conditions

*A. thaliana* (L.) Heynh Col-0 was used. Col *FRI-sf2* (*FRI+*), AT4G00650, was kindly provided by Dr J. Jarillo (Madrid) (Lee and Amasino, 1995). The *flc-6* (SALK_41126, AT5G10140) and *vin3-5* (SALK_004766, AT5G57380) alleles were described previously (Mylne *et al.*, 2006; Bouveret *et al.*, 2006).

### In vitro *plant regeneration*

Seeds were placed on 0.5× Murashige and Skoog (MS) plates and kept in the dark at 4 °C for 4 days. Non-vernalized parent plants were grown under long-day conditions (LD) of 16/8h (light/dark) at 22 ± 2 °C for 4–5 days. For vernalized parent plants, after being kept in the dark at 4 °C for 4 days, plates containing imbibed seeds were moved to short-day conditions (SD) of 8/16h for germination and kept for 6 weeks at 4 °C in the same conditions. After the vernalization treatment, plants were transferred to LD at 22 °C and grown for a further 4–5 days. Excised roots from 4–5-day-old plants (+6 weeks for vernalized plants) were transferred to callus induction medium (CIM) [MS salts (Murashige and Skoog, 1962), 0.1% 2-(*N*-morpholino)ethanesulfonic acid (MES), 1% sucrose, 0.5 mg/l 2,4-dichlorophenoxyacetic acid (2,4-D) (D0911; Duchefa, Haarlem, The Netherlands), 0.1 mg/; kinetin (K2751; Sigma, St. Louis, MO, USA) and 0.8% agarose]. Root explants were cultured on CIM plates under low-light LD (3 µmol m^−2^ s^−1^) for 4–5 days. For shoot induction, root explants that had been cultured in CIM for 4–5 days were transferred to shoot induction medium (SIM) [MS salts, 0.1% MES, 1% sucrose, 0.5 mg/l 6-benzylaminopurine (B0904; Duchefa), 0.125 µM α-naphthalene acetic acid (N0903; Duchefa), 0.75% agarose]. After approximately 1 month of culture on SIM plates under low-light LD, newly emerged shoots were isolated and transferred to plates without any plant growth regulators (1× MS salts, 0.1% MES, 2% sucrose, 0.7% agarose). Flowering date was counted from the start of CIM culture of explants.

### Chromatin immunoprecipitation and expression analysis

For analysis of H3K27me3 levels of regenerated shoot, root tissues and aerial tissues were harvested from 9–10-day-old seedlings (+6 weeks for vernalized plants) as the controls. Regenerated shoots were harvested 5–6 weeks after the start of explantation. For analysis of H3K27me3 levels of prolonged culture callus, root tissues were harvested from 7–9-week-old plants (+6 weeks for vernalized plants) as the controls. Chromatin immunoprecipitation (ChIP) was performed as described (Shu *et al.*, 2014). Rabbit IgG (#i5006; Sigma-Aldrich, St. Louis, MO, USA), anti-H3K27me3 (#07-449; Millipore, Billerica, MA, USA) and anti-histone H3-CT-pan (#07-690; Upstate/Millipore#07-690) antibodies were used. ChIP was performed in three biological replicates. Enrichment was calculated relative to histone H3 for ChIP analysis. Expression analysis was done as described (Maksimov *et al.*, 2016). Gene expression values are given relative to a *PP2a* reference gene (*AT1G13320*). Primers used are listed in Supplementary Table S1 at *JXB* online.

## Results and discussion

### Genetic dissection of flowering time in regenerated shoots derived from vernalized plants

**Fig. 1.**
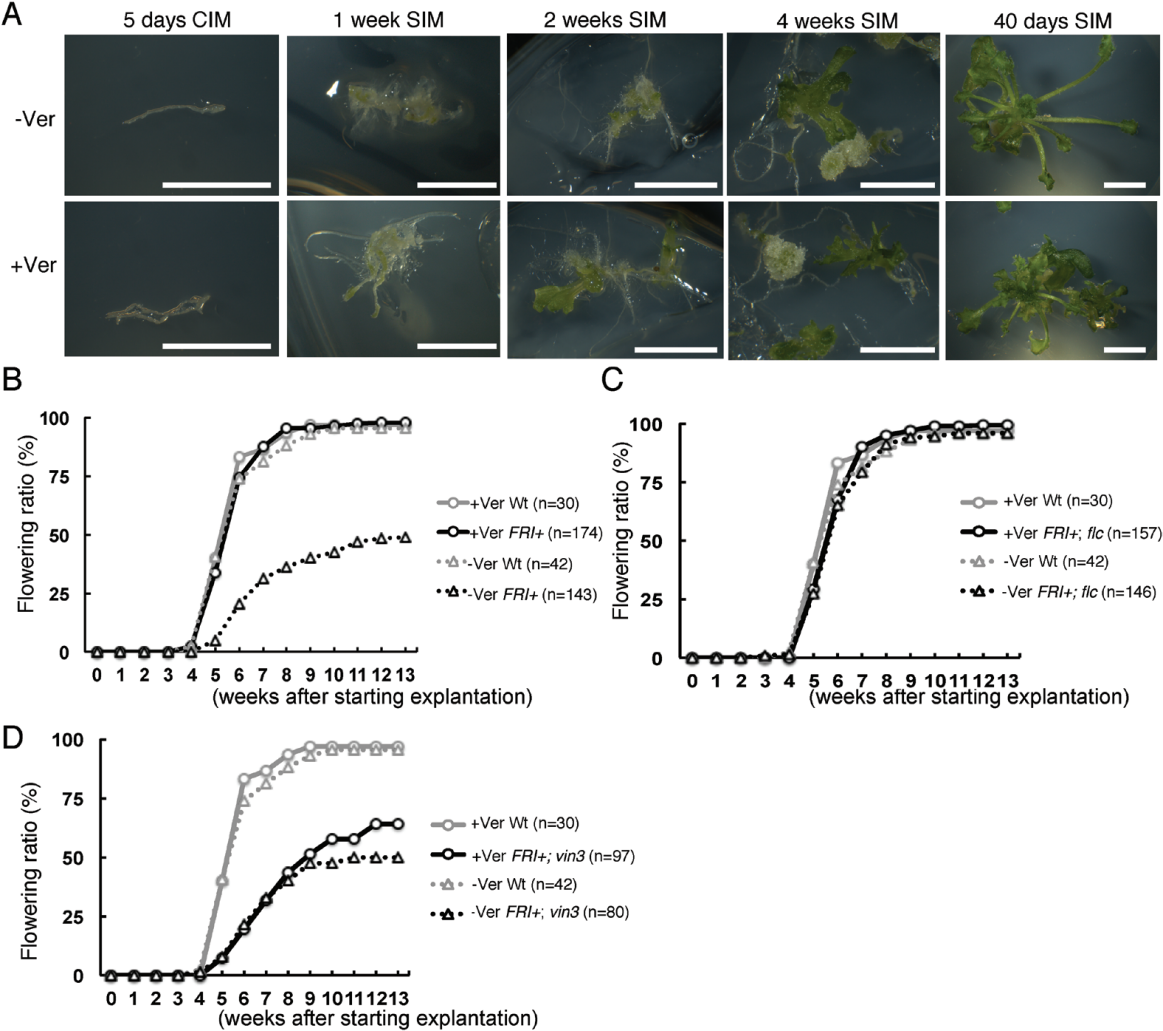
Vernalization effect in regenerated shoots. (A) Tissues during regeneration of non-vernalized or vernalized plants. Images were taken at 5 days after transfer to CIM and at 1, 2, and 4 weeks and 40 days after transfer to SIM. Bars=5 mm. (B–D) Cumulative flowering ratio (%) of regenerated shoots with increasing time after the start of explantation (transfer to CIM). Data for wild-type and (B) *FRI+*, (C) *FRI+;flc*, and (D) *FRI+;vin3*, with or without vernalization, are presented. +Ver, regeneration from vernalized parents; -Ver, regeneration from non-vernalized parents.

Dedifferentiation of plant cells, as occurs during callus formation, causes major chromatin reorganization (Zhao *et al.*, 2001; Williams *et al.*, 2003; Tessadori *et al.*, 2007; He *et al.*, 2012), which could impair the stability of epigenetic states during *in vitro* regeneration. To probe the stability of the vernalized state during *in vitro* regeneration in the Arabidopsis reference strain Col-0, we measured the flowering time of plants regenerated from vernalized or non-vernalized Arabidopsis Col-0 parent plants carrying an active allele of the *FLC* activator *FRIGIDA* (hereafter, *FRI+*), which confers vernalization requirement (Michaels and Amasino, 1999). Roots from *FRI+* seedlings grown at 22 °C for 4–5 days followed by 6 weeks at 4 °C were induced to form callus on CIM. Shoot induction was initiated by transferring the tissue to SIM. Although vernalized plants were 6 weeks older than non-vernalized plants, callus induction and shoot regeneration occurred in a similar manner (Fig. 1A). The number of leaves formed before flowering is often used as a measure of flowering time, but because after *in vitro* regeneration multiple shoots tend to be clustered, leaf number cannot easily be assigned to a particular shoot. Therefore, we used the number of days from transfer to CIM until flowering as a more objective measure of flowering time. After *in vitro* regeneration, wild-type plants and vernalized *FRI+* plants began to flower 3–5 weeks after starting tissue culture (Fig. 1B). Flowering regenerated shoots from vernalized *FRI+* plants had relatively smaller rosette leaves, which is commonly observed in early-flowering plants (Supplementary Fig. S1A–C). In contrast, non-vernalized *FRI+* plants flowered considerably later. Although nearly half of the non-vernalized *FRI+* plants flowered at between 5 and 9 weeks after starting tissue culture, the remaining plants did not flower even at 12 weeks (Fig. 1B). Regenerated shoots of *FRI+* derived from non-vernalized plants had a typical late-flowering phenotype, for example, having excess rosette leaf growth (Supplementary Fig. S1D–F).

Next, we tested whether the late-flowering phenotype in non-vernalized *FRI+* plants depends on the major flowering repressor *FLC* by using *FRI+;flc* parents. Regenerated plants from vernalized and non-vernalized *FRI+;flc* parents flowered similarly to regenerated plants from the wild-type Col-0, which lacks functional *FRI* alleles, or vernalized *FRI+* parents (Fig. 1B, C). This result indicates that *FLC* is required for the late flowering of regenerated plants in the *FRI+* genetic background.

Flowering is favored not only by vernalization but also by other external and internal cues, including plant age (Wu and Poethig, 2006; Wu *et al.*, 2009). Because vernalized plants were 6 weeks older than non-vernalized plants, flowering of plants regenerated from vernalized parents might be facilitated by age. To test whether age or vernalization contributed more to flowering time after regeneration, we measured flowering time after regeneration from a *FRI+;vin3* mutant parent, which lacks the vernalization response but has no known effect on aging (Sung and Amasino, 2004). Similar to shoots derived from non-vernalized *FRI+* parents, shoots regenerated from both vernalized and non-vernalized *FRI+;vin3* parents flowered late (Fig. 1D). This result indicates that vernalization but not age of parents accelerated flowering after regeneration. We noted that approximately half of the shoots regenerated from *FRI+;vin3* plants flowered early (5–9 weeks), regardless of vernalization. This was reminiscent of the early flowering of half of the shoots from non-vernalized *FRI+* plants.

Taking these results together, the effect of vernalization on flowering was stably maintained through *in vitro* regeneration of Arabidopsis Col-0 *FRI+* and depended strictly on *FLC* and *VIN3*.

### *Maintenance of H3K27me3 on* FLC *during dedifferentiation and redifferentiation*

**Fig. 2.**
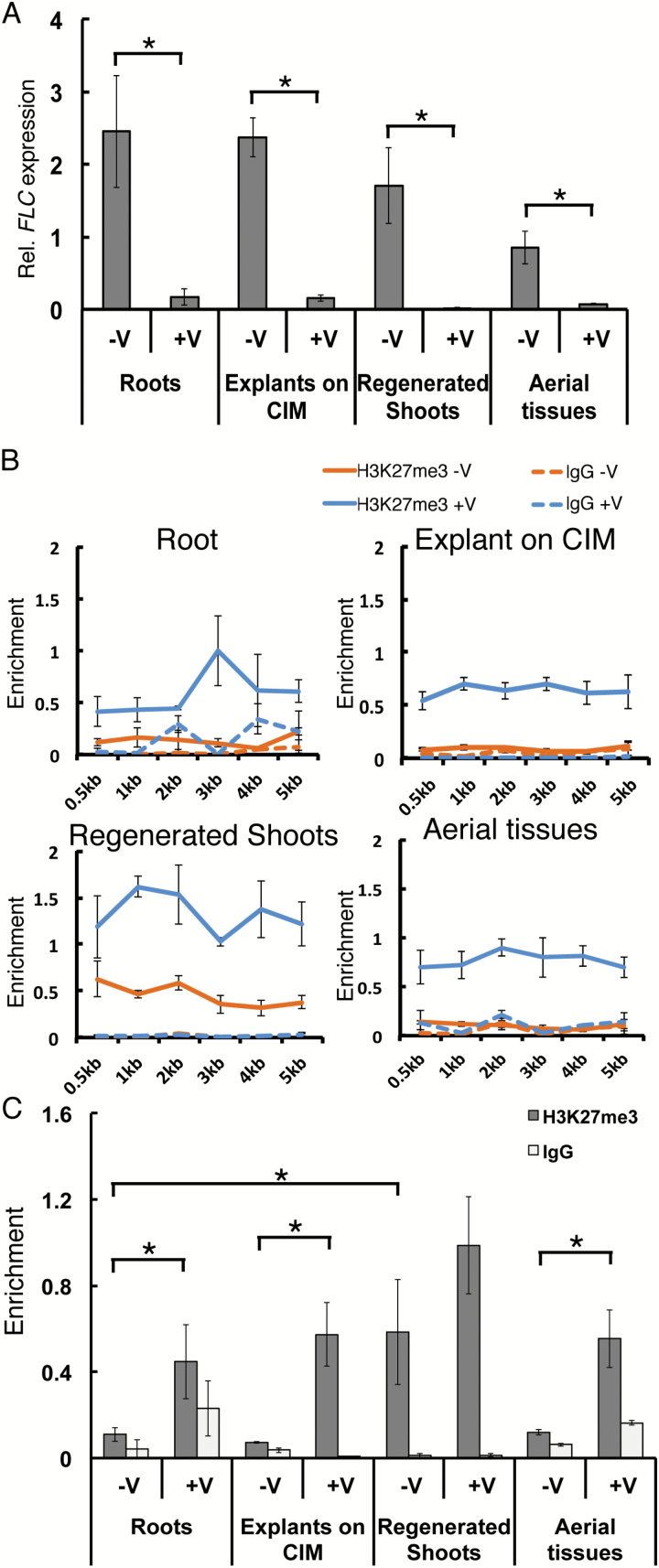
Expression and H3K27me3 levels of *FLC* during shoot regeneration. (A) *FLC* expression in roots, root explants 5 days after transfer to CIM, regenerated shoots, and aerial tissues from regular seed-derived plants of equal age to the root material. Expression is shown relative to that of a *PP2a* control gene. Data are mean±SEM from three biological replicates. *P<0.05 (one-tailed Student’s *t*-test). (B) H3K27me3 levels along *FLC* in the same tissue samples as in A. Data are mean±SEM from three technical replicates of a representative experiment. Three independent ChIP experiments were conducted. (C) H3K27me3 levels at *FLC* measured at the 0.5 kb position in B. +V, regeneration from vernalized parents; -V, regeneration from non-vernalized parents.

The stability of the vernalization effect through *in vitro* regeneration and the requirement for *FLC* suggested that repression of *FLC* by vernalization is maintained through regeneration. To test this hypothesis, we measured *FLC* expression at different stages during regeneration. The regeneration process can be separated into dedifferentiation and redifferentiation, corresponding to callus formation on CIM and shoot generation on SIM, respectively. We used roots cultured for 5 days on CIM as dedifferentiated tissues and regenerated shoots from callus as redifferentiated tissues. All vernalized roots and aerial tissues as well as regenerated shoots from vernalized *FRI+* plants had low *FLC* expression (Fig. 2A). In contrast, *FLC* expression was high in all samples derived from non-vernalized *FRI+* plants. Thus, the activity state of *FLC* is largely maintained through *in vitro* regeneration.

H3K27me3 is required for the maintenance of *FLC* silencing in somatic tissue (Sung and Amasino, 2004), but its stability during *in vitro* culture and regeneration is not known. In animals, undifferentiated cells or induced reprogrammed cells often have reduced H3K27me3 (Mansour *et al.*, 2012; Zhu *et al.*, 2013). Similarly, some plant PcG target genes show major changes of H3K27me3 levels during callus induction (He *et al.*, 2012). To test whether H3K27me3 levels at *FLC* change during dedifferentiation and redifferentiation, we performed ChIP. Across the *FLC* gene body, relative H3K27me3 levels were maintained throughout regeneration in both vernalized and non-vernalized samples (Fig. 2B). H3K27me3 accumulates at the *FLC* gene with distinct spatial dynamics. During cold exposure, the nucleation region (from the transcription start site to the 1 kb region) and the distal region (near the 4–5 kb region) gradually gain H3K27me3 (Angel *et al.*, 2011). After moving to warm temperatures, H3K27me3 spreads over the entire *FLC* gene (Angel *et al.*, 2011). In regenerated shoots, a 3 kb region located between the nucleation and distal regions had slightly lower H3K27me3 levels than other regions (Fig. 2B). Given this pattern, the increase in H3K27me3 during *in vitro* culture might occur through a similar molecular mechanism to that induced by vernalization. Considering that H3K27me3 is a repressive mark, this result is consistent with the observed expression of *FLC*. Taken together, these results demonstrate that *FLC* repression and the presence of H3K27me3 are heritable throughout the *in vitro* regeneration process. In addition, the regeneration process favored some H3K27me3 accumulation at *FLC* even in regenerated shoots from non-vernalized plants (Fig. 2C).

The histone demethylase EARLY FLOWERING 6 (ELF6) contributes to the epigenetic resetting of *FLC* during sexual reproduction (Crevillén *et al.*, 2014). We tested whether the lack of epigenetic resetting during *in vitro* regeneration was caused by lack of expression of *ELF6* or its homolog *RELATED TO ELF6* (*REF6*) during *in vitro* regeneration (Supplementary Fig. S2). However, both *ELF6* and *REF6* were expressed at similar levels in ovules and in callus samples, suggesting that expression of the two H3K27me3 demethylase genes is not sufficient for epigenetic resetting of *FLC*. Instead, demethylase targeting or activity might not be appropriate for *FLC* resetting during *in vitro* regeneration. Interestingly, *REF6* expression increased in callus samples (Supplementary Fig. S2). This is consistent with widespread changes in H3K27me3 during dedifferentiation (He *et al.*, 2012), which, however, seem not to affect *FLC*.

### FLC *is repressed in some shoots derived from non-vernalized parents*

**Fig. 3.**
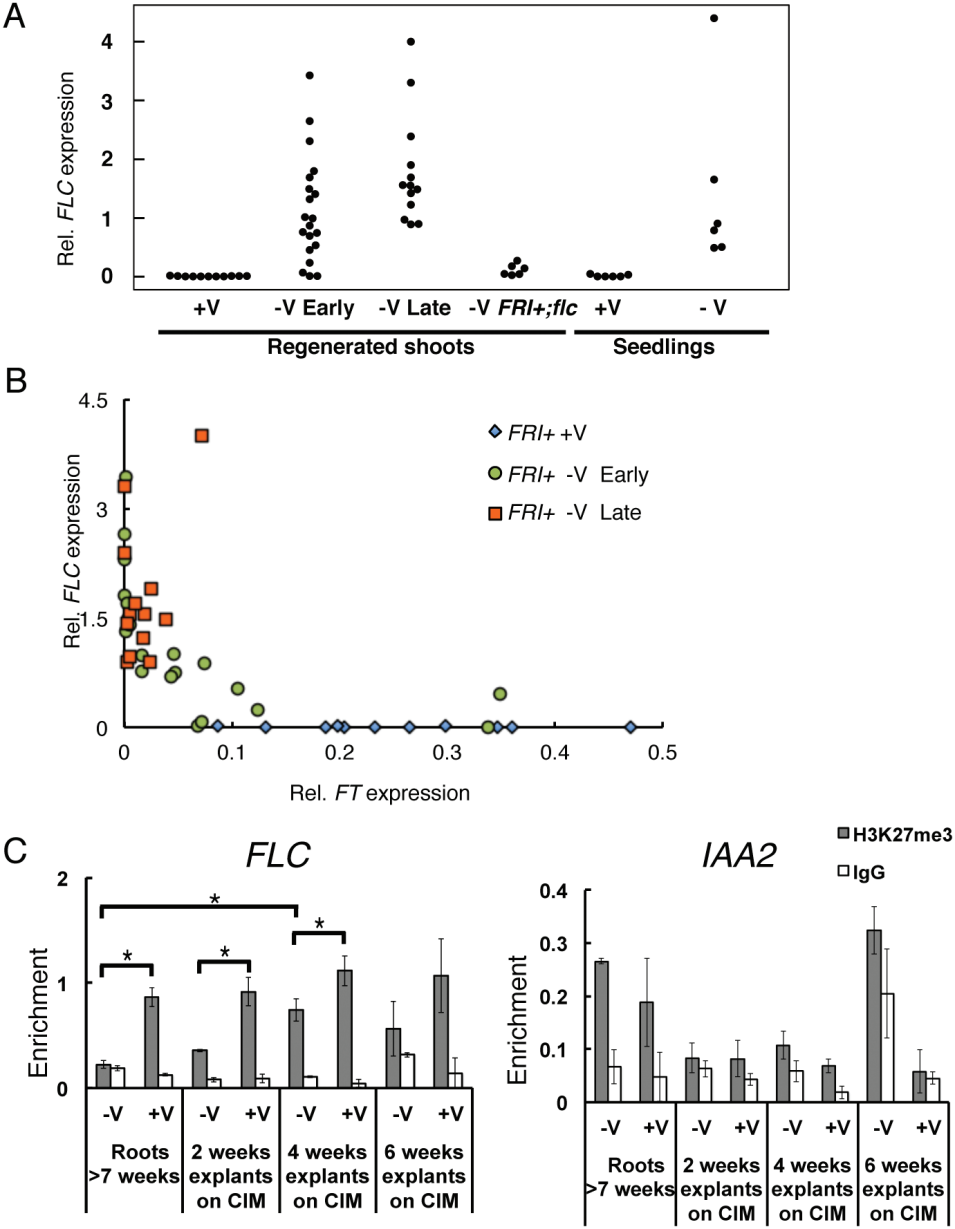
Effects of prolonged *in vitro* culture and of regeneration on *FLC* expression and H3K27me3 levels. (A) *FLC* expression in individual regenerated shoots in the *FRI+* background. +V, regeneration from vernalized parents; -V, regeneration from non-vernalized parents; Early, early-flowering plant (flowering at sampling); Late, late-flowering plant (not flowering at sampling). (B) Relationship between *FLC* and *FT* expression in leaves of shoots regenerated from *FRI+* parents. Blue diamonds, shoots derived from vernalized *FRI+* parents; green circles, early-flowering shoots derived from non-vernalized *FRI+* parents; orange squares, late-flowering shoots derived from non-vernalized *FRI+* parents. (C) Effect of prolonged CIM culture on the H3K27me3 levels on *FLC* and the control locus *IAA2*. Data are mean±SEM from three technical replicates of a representative experiment. Three independent ChIP experiments were conducted. *P<0.05 (one-tailed Student’s *t*-test).

When pooled samples were used for mRNA expression analysis, no obvious suppression of *FLC* was observed (Fig. 2A). However, about half of the regenerated shoots that were derived from non-vernalized *FRI+* parents flowered before 12 weeks after transfer to CIM (Fig. 1B), suggesting that *FLC* expression may vary among individual regenerated plants from non-vernalized parents. To test this hypothesis, we measured *FLC* expression in individual regenerated shoots that were either early or late flowering (Fig. 3A). As expected, all shoots derived from vernalized *FRI+* parents had very low *FLC* expression. Late-flowering shoots that were derived from non-vernalized parents had relatively high *FLC* expression. In contrast, some early-flowering shoots that were derived from non-vernalized parents had *FLC* expression as low as shoots from vernalized *FRI+* plants (Fig. 3A). This suggested that *in vitro* regeneration from non-vernalized parents has the potential to stochastically influence *FLC* expression.

Interestingly, a moderate increase of H3K27me3 at *FLC* was observed in regenerated shoots that were derived from non-vernalized or vernalized parents (Fig. 2C). Considering the correlation between the stochastic suppression of *FLC* expression and the partial increase of H3K27me3, the regeneration process could initiate stochastic alteration of the active *FLC* state through histone modification.

Because some shoots from non-vernalized plants flowered early despite high *FLC* expression, *FLC* expression and flowering time were not strictly correlated in regenerated shoots (Fig. 3A). It was possible that flowering despite high *FLC* levels was caused by independent activity of the floral activator *FT*. However, we observed a clear anti-correlation pattern between *FLC* and *FT* expression in all tested shoots (Fig. 3B). *FT* was expressed in shoots derived from non-vernalized *FRI+* parents only when *FLC* expression was low. Unexpectedly, most of the early-flowering shoots from non-vernalized parents had low *FT* expression, suggesting that flowering in these regenerated plants occurred independent of *FT*. The stochastic suppression of *FLC* and apparently *FT*-independent flowering were also observed in *FRI+;vin3* (Supplementary Fig. S3A, B). Further work will be needed to elucidate the mechanism of flowering in the presence of *FLC* after regeneration. Together, these findings indicate that the inactive, but not the active, epigenetic state of *FLC* is stably retained through *in vitro* regeneration.

### *Effect of prolonged* in vitro *culture on the H3K27me3 level at* FLC

Long-term callus culture can induce hormone-independent cell proliferation, a process termed habituation (Gautheret, 1955). In habituated callus, potential epigenetic clonal alterations occur and the transcription levels of epigenetic regulators can change (Binns and Meins, 1973; Pischke *et al.*, 2006). While the 5–7 days of in *vitro* culture before shoot induction in our original protocol did not affect the inactive epigenetic state of *FLC*, prolonged *in vitro* culture might alter the inactive epigenetic state of *FLC*. To test this hypothesis, we measured H3K27me3 levels at *FLC* in calli that were cultured for 2, 4, and 6 weeks on CIM. H3K27me3 at *FLC* was always higher in calli derived from vernalized plants than in calli from non-vernalized plants (Fig. 3C). However, for 4- and 6-week-old calli derived from non-vernalized plants, H3K27me3 levels were slightly but reproducibly higher than in 7-week-old roots, which have the same total age at 22 °C as 6-week-old calli. As a control, we also measured H3K27me3 levels on *IAA2*, which has high H3K27me3 in leaves but not in callus (Fig. 3C) (He *et al.*, 2012). H3K27me3 signals at *IAA2* showed no clear difference compared with IgG controls during CIM culture, suggesting that the *IAA2* locus maintained low H3K27me3 levels throughout 6 weeks of *in vitro* culture on CIM, in contrast to *FLC.* Thus, callus induction and extended culture on CIM can cause a vernalization-independent increase of H3K27me3 at the *FLC* locus.

In conclusion, our study reveals that the silenced, H3K27me3-positive epigenetic state of *FLC* after vernalization, which is reset during sexual reproduction, is stably maintained during *in vitro* regeneration. In contrast, the active epigenetic state of *FLC* is only partially maintained through *in vitro* reproduction and can stochastically convert to the silent state.

## Supplementary data

Supplementary data are available at *JXB* online.

Fig. S1. Plant morphology of shoots regenerated from *FRI+* parents.

Fig. S2. Expression of histone demethylases *REF6*, *ELF6*, and *FLC* during shoot regeneration.

Fig. S3. *FLC* expression in leaves from *FRI+;vin3* regenerated shoots.

Table S1. Primer sequences in this study.

## Author contributions

MN performed the experiments and analyzed data. MN and LH conceived and designed the experiments and wrote the manuscript.

## Supplementary Material

Supplementary_Table_S1_Figures_S1_S3
