# Supplementary material for: Inheritance of vernalization memory at FLOWERING LOCUS C during plant regeneration
Source: J Exp Bot. 2017 May 11;68(11):2813–9. doi: 10.1093/jxb/erx154 (PMC5853441; doi:10.1093/jxb/erx154)
Supplement: Supplementary_Table_S1_Figures_S1_S3 [file erx154_suppl_supplementary_table_s1_figures_s1_s3.pdf]

Table S1. Primer sequences in this study

Target region	Primer for ChIP-qPCR	Sequence (5′- -3′)
*FLC*_0.5kb	a_MD001	atctcttgttgtttctcggttctg
	a_MD008	tcactcaacaacatcgagcac
*FLC*_1kb	LH1815	cacagtagttttgaattttggtagctt
	LH1816	tgaagtagcatatgtgcggtaag
*FLC*_2kb	LH3574	agccttttagaacgtggaacc
	LH3575	tcttccatagaaggaagcgact
*FLC*_3kb	LH671	tcttaaatgcaaacaagcaatttt
	LH670	tcggtttacttccatgtcatca
*FLC*_4kb	LH1771	cgcaattttcatagcccttg
	LH1772	ctttgtaatcaaaggtggagagc
*FLC*_5kb	LH1811	ttccggttgttggacataact
	LH1812	tctccaaatgaaccaaaacctaa
IAA2 (AT3G23030)	LH3202	gtacgagaaagtcaacgagc
	LH3203	acgcaagaaacctcttgttc

Target region	Primer for RT-qPCR	Sequence (5′- -3′)
FLC	LH0304	gactgccctctccgtgacta
	LH0305	ttctcaacaagcttcaacatgag
PP2a *(AT1G13320)*	LH308	ggagagtgacttggttgagca
	LH309	cattcaccagctgaaagtcg
FT (AT1G65480)	LH0312	ggtggagaagacctcaggaa
	LH0313	ggttgctaggacttggaacatc
REF6 (AT3G48430)	LH1708 (Deng et al 2007)	cggaataccgtgttgcaggttag
	LH1709 (Deng et al 2007)	ccggatagcagcatctttagcca
ELF6 (AT5G04240)	LH3010	aattccttgttatttgccataaaga
	LH3011	catcatagctgaaagggactttg

Deng, W., Liu, C., Pei, Y., Deng, X., Niu, L. and Cao, X. 2007. Involvement of the histone acetyltransferase AtHAC1 in the regulation of flowering time via repression of FLOWERING LOCUS C in Arabidopsis. Plant Physiology. 143**,** 1660– 1668.

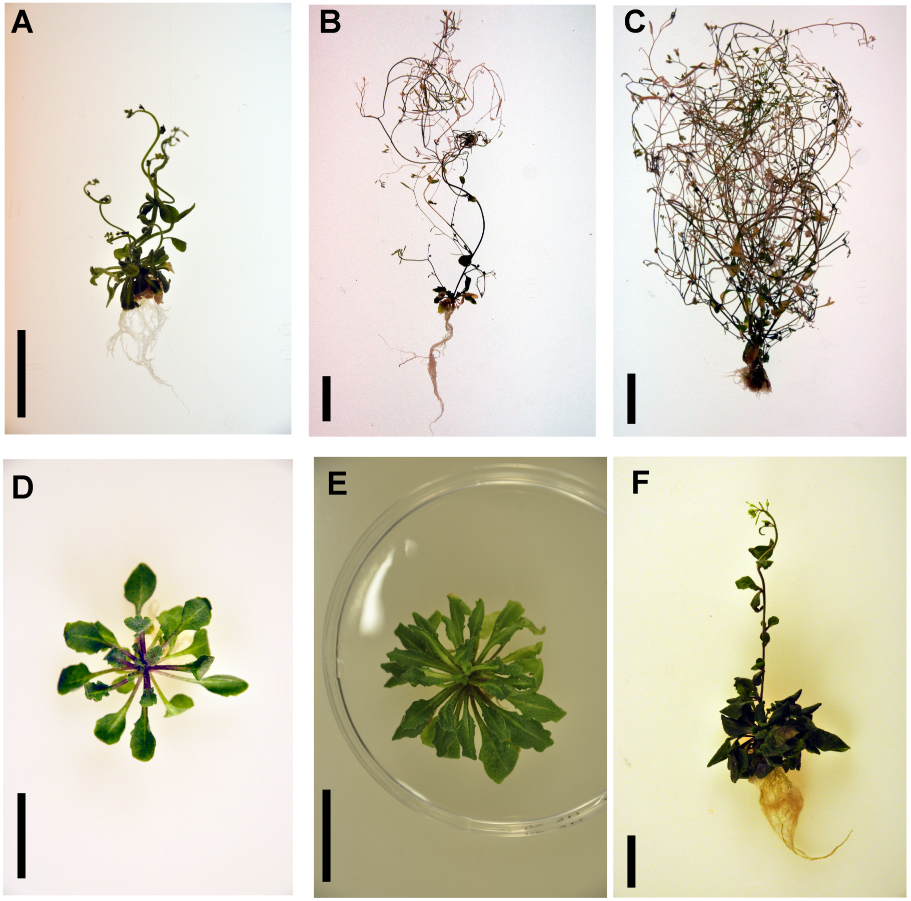

Fig. S1. Plant morphology of regenerated shoots in the *FRI*+ background. (A-C) Shoots that were derived from vernalized parental plants. (D-F) Shoots that were derived from nonvernalized parental plants. (A, D) 6 weeks after starting explantation. (B, C, E, F) Approximately 12 weeks after starting explantation. Scale bars =20 mm

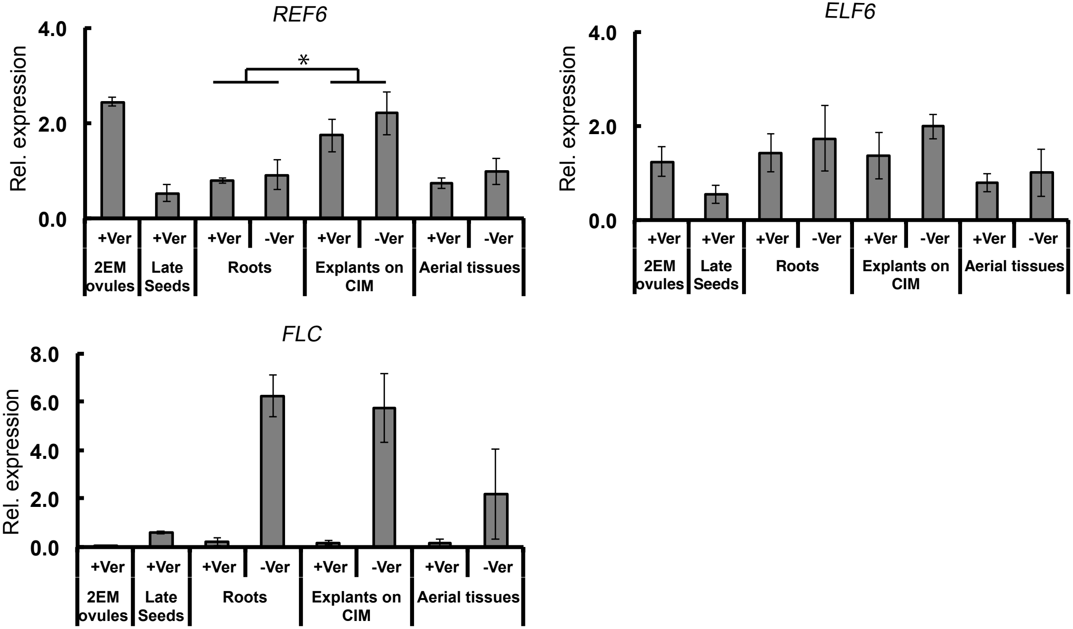

Figure S2. Expression of histone demethylases *REF6*, *ELF6* and *FLC* during shoot regeneration.

Relative expression values are mean ± SEM from 2 biological replicas of ovules and late embryos and from 3 biological replicas of roots, explants and aerial tissues. Asterisk indicates a significant difference in the one-tailed Student's *t*-test ($p < 0.05$).

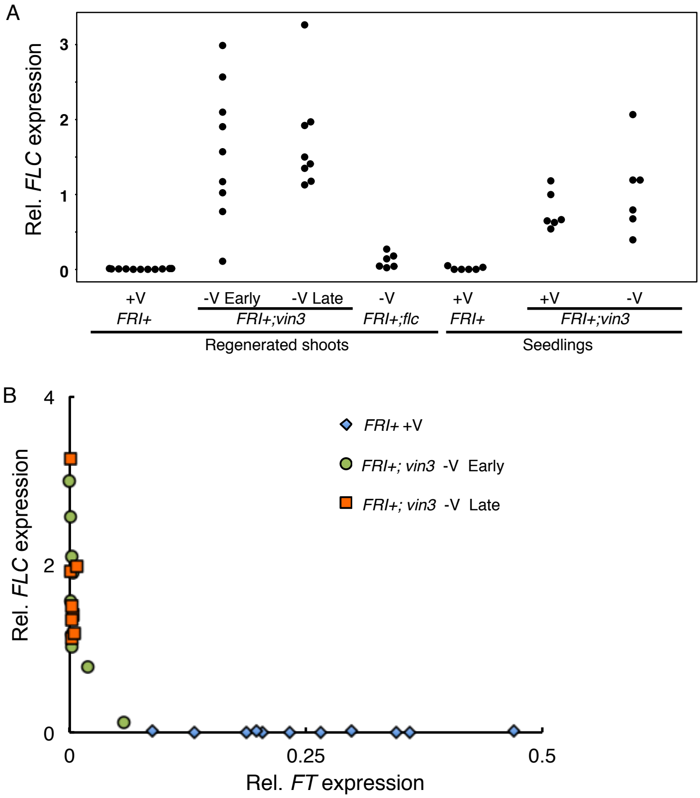

Figure S3. *FLC* expression in leaves from *FRI+; vin3* regenerated shoots. (A) *FLC* expression in individual regenerated shoots in the *FRI+;vin3* background. +V regeneration from vernalized parents. *FRI+* and *FRI+;flc* are control. -V, regeneration from non-vernalized parents. Early: early flowering plants (flowering at sampling), Late: late flowering plants (not flowering at sampling). (B) Relationship between *FLC* and *FT* expression in leaves from shoots regenerated from *FRI+* or *FRI+;vin3* parents. Blue diamonds: shoots derived from vernalized *FRI+* parents. Green circles: early flowering shoots derived from non-vernalized *FRI+;vin3* parents. Orange squares: late flowering shoots derived from non-vernalized *FRI+;vin3* parents.
